# Extracellular histones are a target in myocardial ischaemia–reperfusion injury

**DOI:** 10.1093/cvr/cvab139

**Published:** 2021-04-20

**Authors:** Mohammed Shah, Zhenhe He, Ali Rauf, Siavash Beikoghli Kalkhoran, Christina Mathisen Heiestad, Kåre-Olav Stensløkken, Christopher R Parish, Oliver Soehnlein, Sapna Arjun, Sean M Davidson, Derek Yellon

**Affiliations:** 1 The Hatter Cardiovascular Institute, Institute of Cardiovascular Science, University College London, 67 Chenies Mews, London, WC1E 6HX, UK; 2 Section of Physiology, Department of Molecular Medicine, Institute for Basic Medical Sciences, University of Oslo, Oslo, Norway; 3 ACRF Department of Cancer Biology and Therapeutics, The John Curtin School of Medical Research, The Australian National University, Canberra, ACT, Australia; 4 Institute for Cardiovascular Prevention (IPEK), LMU Munich Hospital, Pettenkoferstrasse 8a, D-80336 Munich, German y; 5 Institute for Experimental Pathology (ExPat), Center for Molecular Biology of Inflammation, WWU Münster, Von-Esmarch-Strasse 56 48149 Münster, German y; 6 Department of Physiology and Pharmacology (FyFa), Karolinska Institutet, Stockholm, Sweden

**Keywords:** Histones, Ischaemia, Reperfusion, DAMPs, Cardiomyocyte death

## Abstract

**Aims:**

Acute myocardial infarction causes lethal cardiomyocyte injury during ischaemia and reperfusion (I/R). Histones have been described as important Danger Associated Molecular Proteins (DAMPs) in sepsis. The objective of this study was to establish whether extracellular histone release contributes to myocardial infarction.

**Methods and results:**

Isolated, perfused rat hearts were subject to I/R. Nucleosomes and histone-H4 release was detected early during reperfusion. Sodium-β-O-Methyl cellobioside sulfate (mCBS), a newly developed histone-neutralizing compound, significantly reduced infarct size whilst also reducing the detectable levels of histones. Histones were directly toxic to primary adult rat cardiomyocytes *in vitro.* This was prevented by mCBS or HIPe, a recently described, histone-H4 neutralizing peptide, but not by an inhibitor of TLR4, a receptor previously reported to be involved in DAMP-mediated cytotoxicity. Furthermore, TLR4-reporter HEK293 cells revealed that cytotoxicity of histone H4 was independent of TLR4 and NF-κB. In an *in vivo* rat model of I/R, HIPe significantly reduced infarct size.

**Conclusion:**

Histones released from the myocardium are cytotoxic to cardiomyocytes, via a TLR4-independent mechanism. The targeting of extracellular histones provides a novel opportunity to limit cardiomyocyte death during I/R injury of the myocardium.

## Graphical Abstract

**Figure:**
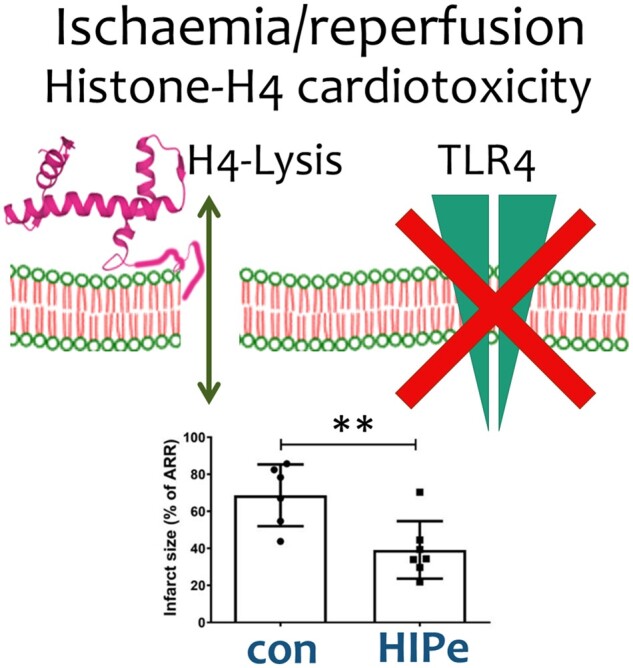

**Translational perspective**

Acute myocardial infarction causes lethal cardiomyocyte injury during ischaemia and reperfusion (I/R). New approaches are needed to prevent cardiomyocyte injury and limit final infarct size. We show that histones released from damaged cells, and histone-H4 in particular, causes rapid cardiomyocyte death during I/R. mCBS, a compound targeting histones non-specifically, was cardioprotective in *ex vivo* rat hearts, while HIPe, a targeting histone H4 specifically, was cardioprotective in an *in vivo* rat model. HIPe may have potential as a therapeutic agent in the setting of acute myocardial infarction.

## 1. Introduction

Acute myocardial infarction (MI) is a major cause of death and disability worldwide.^1^ Timely reperfusion is important to limit acute myocardial injury, but nevertheless results in substantial ischaemia and reperfusion (I/R) injury. Therefore, novel approaches are needed to limit I/R injury. Danger Associated Molecular Proteins (DAMPs) are molecules that are released from cells that can activate a sterile immune response, for example, via stimulating Toll-like receptor 4 (TLR4). Following I/R, necrotic debris released from dead cells act as DAMPs, and contribute to excessive cardiomyocyte death and inflammation.^2^^,^^3^ DAMPs include fragmented nuclear and mitochondrial DNA.^4^ Inside the cell, nuclear DNA is packaged in nucleosomes consisting of superhelical DNA wound around an octamer of histones, which are composed of two of each of the core histones H2A, H2B, H3 and H4, plus one linker histone H1.^5^ Histones are also now known to be major DAMPs, a fact first demonstrated by the observation that mice rapidly die when free histones are administered intravenously.^6^ In an ischaemic stroke model, histone infusion increased brain infarct size and conversely, histone neutralization via antibody infusion reduced infarct size.^7^ Similar cytotoxic effects of histones have been demonstrated in kidney injury,^8^ sepsis,^6^ and even hair follicle death.^9^ In particular, histone H4 has recently been identified as the toxic mediator of smooth muscle cell death in a mouse model of atherosclerosis.^10^ The cytotoxic effect of extracellular histones suggests they may represent a potential target for limiting myocardial I/R injury.^4^

It has previously been reported that histones activate TLR4^8^^,^^11^ and may be implicated in the activation of regulated cell death processes in the cardiovascular system. Myocardial I/R is associated with regulated cell death mechanisms such as caspase-dependent pyroptosis.^12^ Unlike apoptosis, pyroptosis results in cell lysis associated with the release of large quantities of cellular DNA in the infarct zone.^9^^,^^13^ A second source of extracellular histones and DNA is neutrophils that undergo NETosis, releasing neutrophil extracellular traps (NETs), which consist of granule proteins and chromatin structures that are rich in histones.^14^ NETs play a crucial role in thrombosis, platelet aggregation^15^ and blood vessel occlusion, and can thereby further exaggerate coronary ischaemia. Thrombi aspirated from the coronary arteries of patients who suffered ST-elevation MI demonstrate that the burden of NETosis positively correlates with infarct size and negatively correlates with ST-segment resolution.^16^ Extracellular histones, therefore, exist in two states: as NET/chromatin-associated histones, and as free histones that are released from NETs/chromatin by either endogenous or exogenous nucleases. Consequently, histones from these two sources might contribute to myocardial injury during I/R.

Histone antagonists, which are designed to neutralize histones and suppress their inflammatory signalling, may be an important means of protecting against I/R injury.^17^ Extracellular histones are highly cationic due to the large number of exposed basic residues on their surface. Therefore, polyanions such as heparin can interact electrostatically with histones and neutralize them.^18^ However, heparin’s large molecular structure means this pleiotropic action would only be effective in potentially lethal doses, as a result of its considerable anti-coagulant property. Non-anticoagulant heparins have also demonstrated effective histone neutralizing activities. One recently developed non-anticoagulant polyanion is sodium-β-O-Methyl cellobioside sulfate (mCBS). Its small, polyanionic structure allows it to reduce histone-induced systemic toxicity, as shown in pharmacodynamics studies in mouse and rabbit, and it can be administered in much higher doses than heparin without any anticoagulant effect. It has also shown a greater affinity to extracellular histones in comparison to non-anticoagulant heparins.^19^^,^^20^

Recently, a more specific, cyclic peptide called HIPe has been developed, which selectively binds to the N terminus of histone H4, disrupting its interaction with cell membranes thereby preventing its lethal effect.^10^ In a recent letter to Nature, HIPe was shown to neutralize histone H4 and prevent histone H4-mediated death of smooth muscle cells in mouse models of atherosclerosis.^10^

Based on the above, we hypothesized that the release of extracellular histones may contribute to excessive cardiomyocyte death during myocardial I/R injury. Furthermore, inhibiting histones with mCBS or inhibiting histone H4 specifically with HIPe could protect the heart from the damaging effects of I/R, potentially by preventing the stimulation of TLR4.

## 2. Methods

### 2.1 Ethical approval

The animal experiments were conducted within the terms of the Animals (Scientific Procedures) Act 1986, under Project Licence number PPL 70/8556, (‘Protection of the Ischaemic and Reperfused Myocardium’) issued to Prof. Derek Yellon in 2015. All procedures conform to the guidelines from Directive 2010/63/EU of the European Parliament on the protection of animals used for scientific purposes.

### 2.2 Animals

All the animals used throughout were male Sprague–Dawley (SD) rats weighing 300–400 g and were bred at UCL. Rats were obtained from the animal handling unit on the day of the experiment. The rats were humanely restrained in a plastic cone and subjected to an intraperitoneal injection of 90 mg/kg of 20% Pentobarbitone. The operator made regular checks on respiratory rate and general alertness. The animals were deemed sedated once hind limb and corneal reflexes could no longer be elicited. The chest cavity was opened via a clamshell thoracotomy and the rats were euthanized by severing the aorta. The heart was immediately removed and placed in ice cold Krebs Henseleit Buffer (KHB).

### 2.3 Krebs Henseleit buffer

KHB, prepared fresh each day, consisted of 118 mM NaCl, 25 mM NaHCO_3_, 11 mM d-glucose, 4.7 mM KCl, 1.22 mM MgSO_4_·7H_2_O, 1.21 mM KH_2_PO_4_ and 1.84 mM CaCl_2_, 2H_2_O, pH 7.40.

### 2.4 *Ex-vivo* Langendorff retrograde perfusion of the heart

Once harvested, the isolated rat hearts were transferred to a Langendorff retrograde perfusion apparatus for global I/R as described by Bell *et al*.^21^ Hearts were randomly allocated to receive either KHB, as a control, or mCBS, dissolved in KHB at a concentration of 100 µg/mL, as the treatment arm. Hearts in the treatment arm were initially perfused with standard KHB, 5 min prior to the onset of ischaemia and then switched to the buffer containing the mCBS and perfused with the drug throughout 45 min of global ischaemia and 2 h reperfusion. In some experiments, mCBS was added to the perfusate only during 2 h reperfusion. In some experiments, histones were added to the perfusate at the indicated concentration, beginning 5 min prior to ischaemia and continuing throughout reperfusion. The hearts were then removed, frozen for 15 min and then sliced into 5 mm slices for further analysis. The slices were then histologically stained with 2,3,5-triphenyltetrazolium chloride (TTC) to differentiate viable tissue from infarcted tissue and then subsequently scanned and analysed (using Image J software) to determine the size of the infarct.

### 2.5 Quantification of histones in perfusate

Samples of perfusate (2 mL) were directly collected from the perfused hearts during stabilization, immediately at the point of reperfusion and at 5 min intervals thereafter. The samples were directly frozen at −80°C for analysis of histones at a later date. The perfusate was analysed for the presence of histones using a commercially available ELISA assay (Sigma-Aldrich). The concentration of histone H4 was analysed using a DELFIA (dissociation-enhanced lanthanide fluorescence immunoassay) with antibodies specific to histone H4, as follows. The perfusate samples were thawed at room temperature. 20 μL aliquots of perfusate were added to an ELISA plate that was pre-coated with anti-histone antibody. Following washing, a secondary antibody to DNA was added to the wells, followed by streptavidin-based fluorescence detection. After two hours of incubation followed by multiple washes, fluorescence was analysed with a plate reader at 450 nm wavelength. Values were converted to a concentration by establishing a dose response curve of increasing concentrations of commercially available histones derived from calf thymus.

### 2.6 Isolation of primary adult rat ventricular cardiomyocytes

The isolation buffer contained 130 mM NaCl, 5.4 mM KCI, 1.4 mM MgCl_2_, 0.4 mM Na_2_HPO_4_, 4.2 mM HEPES, 10 mM glucose, 20 mM taurine, and 10 mM creatine, with pH adjusted to 7.4. The process of isolation involved rats being anesthetised, using 0.4 mg/kg phenobarbital, and the heart removed, cannulated and perfused as described above. The heart was then digested by perfusing with isolation buffer containing 0.06% collagenase and 0.01% protease plus 100 µM CaCl_2_. The resultant cardiomyocyte solution then had calcium gradually reintroduced during several buffer washes with 500 µM CaCl_2_ and then 1 mM CaCl_2_ before finally being resuspended in medium 199 (ThermoFisher) supplemented with 5 mM creatine, 2 mM carnitine, 5 mM taurine, 50 units/mL penicillin and 50 µg/mL streptomycin. Cardiomyocytes were seeded in 24-well plates on areas preincubated for at least 1 h with 20–40 μg/mL laminin to facilitate cell adherence. Cardiomyocytes were left to stabilize overnight in a conventional tissue culture incubator at 37°C and 5% CO_2_ before being used further.

### 2.7 *In vitro* treatment of adult rat cardiomyocytes

The primary rat cardiomyocytes, seeded on a 24 well microplate, were treated with vehicle or calf thymus-derived histones (Sigma–Aldrich) dissolved in a phosphate buffer solution and then incubated for 1 h at 37°C and 5% CO_2_. Some cells were additionally exposed to mCBS at a dose of 25 μg/mL and 100 μg/mL (the doses were determined in accordance with pilot data contained in a published patent^19^) 5 μM TAK-242^22^ or the cyclic peptide HIPe peptide^10^ at the indicated concentrations. The cells were then treated with 2 μg/mL propidium iodide (PI) for 15 min after which they were visualized under a fluorescence microscope. PI is a fluorescent DNA intercalating agent that can enter cells via damaged membranes and bind to DNA in the nucleus of cells and fluoresces red, thus providing a marker of cell death. After blinding to treatment, the photographs were analysed using Image J software. Dead cells were defined as those that showed evidence of uptake of PI which was measured as the membrane fluorescence intensity for cardiomyocytes.

As a second marker of cell death, the concentration of lactate dehydrogenase (LDH) released from dead cells was estimated using a commercially available LDH assay kit (Thermo scientific).

### 2.8 TLR4/NF-κB/SEAP reporter HEK293 cells

The commercially available HEK-Blue™ mTLR4 cell line (Invivogen, San Diego, CA, USA), stably transfected with murine TLR4, MD-2, and CD14 co-receptor genes, and an NF-κB-inducible SEAP (secreted embryonic alkaline phosphatase) reporter gene, was used to assess TLR4-dependent NF-κB activity. The parental cell line HEK-Blue Null1-v (Invivogen, San Diego, CA, USA) served as a negative control. Both cell types were cultured according to the manufacturer’s instructions.

Cytotoxicity was measured from the accumulation of formazan, metabolized from 3-(4,5-dimethylthiazol-2-yl)-2,5-diphenyltetrazolium bromide (MTT), by mitochondrial dehydrogenase which is only activated in viable cells. HEK-Blue™ mTLR4- and HEK-Blue Null1-v cells (Invivogen, San Diego, CA, USA) were seeded in a 96-well plate, 4 × 10^4^ cells/well, and treated overnight with mCBS. MTT (Sigma, St. Louis, USA) dissolved in PBS (0.5 mg/mL) and filter sterilized (0.22 µm) (VWR, Pennsylvania, USA) was added to the wells in serum-free cell medium in a 1:10 dilution and incubated for 30 min, at 37°C, in 2% CO_2_. Intracellular formazan was dissolved in dimethyl sulfoxide (DMSO) (Sigma, St. Louis, USA) before absorbance was measured at 550 nm using a BioTek PowerWave XS Microplate Spectrophotometer (BioTek, Vermont, USA).

Detection of NF-kB-induced SEAP production was determined with the use of HEK-Blue™ Detection medium (Invivogen, San Diego, CA, USA) and carried out according to the manufacturer`s protocol. In short, 4 × 10^4^ cells/well were seeded in a 96-well plate. Cells were treated over-night with mCBS and NF-kB activity was indirectly measured with a BioTek PowerWave XS Microplate Spectrophotometer (BioTek, Vermont, USA) at 630 nm, based on the accumulation of hydrolyzed SEAP colour substrate in the medium.

### 2.9 HL-1 cell culture and treatment

The HL-1 cardiac cell line (derived from murine atrial cardiomyocytes) was cultured according to published methods.^23^ Cells were plated at a density of 3 × 10^4^/mL in Cellview coverslip dishes overnight prior to 40 min treatment with vehicle (water), 10 ng/mL TNFα (Abcam) or 10 μg/mL of histones. Cells were then washed with PBS and fixed with 4% paraformaldehyde for 15 min, permeabilized with 0.4% triton-X100 in PBS, blocked in 5% BSA for 1 h, then incubated for 1 h with 1:1000 rabbit anti-NF-kB (Cell Signalling 8242S), followed by 4 µg/mL anti-rabbit Alex Fluo488 (Thermo A11008) and 1:2000 Hoechst 33258 (Thermofisher H3570). Cells were imaged with sequential scans using the 405 nm and 488 nm laser lines of a Leica SP5 confocal microscope and ×63 objective. The percentage of cells with positively stained nuclei was counted in five separate images per experiment.

### 2.10 *In vivo* rat coronary artery occlusion reperfusion model

Rats (280–360 g) were anaesthetised with an initial dose of 100–120 mg/kg pentobarbital. Tracheostomy was performed, and artificial ventilation was achieved by connecting to a Small Animal Ventilator (Harvard Apparatus). 1-lead electrocardiogram (ECG) was recorded using PowerLab 4/30 system (AD Instruments) and LabChart 7 software. Drug or vehicle (PBS) were injected into the vein using a 25 G needle attached to a syringe. Treatments were administered 30 min before ligation of the coronary artery. Thoracotomy was performed and a silk suture was placed underneath the left anterior descending (LAD) artery and coronary artery occlusion was achieved by ligation of the suture. The heart was subjected to 30 min of ischaemia followed by 2 h reperfusion. HIPe (2 mg/kg), mCBS (100 mg/kg), or vehicle (PBS) was given via i.v. injection 10 min before reperfusion. mCBS was alternatively given by bolus (100 mg/kg) followed by infusion throughout I/R at a rate of 0.83 mg/kg/min via jugular vein. Once the protocol was completed, the coronary artery was ligated permanently, the animal was euthanized by severing the aorta and the heart was removed. Evans Blue dye [0.5% w/v in high-K^+^ (30 mM) PBS] was injected to demarcate the non-at-risk area. The heart was then stained and analysed using the methods previously described.

### 2.11 Statistics

Sample size is stated in the figure legends and the data are plotted as individual values and/or means ± SEM. Statistical analyses were performed using Student’s *t*-test for two sample comparisons, or one-way ANOVA followed by Tukey post-test, or two-way ANOVA followed by Bonferroni's correction for multiple comparisons. GraphPad Prism 8.4.2 was used for statistical analyses and graph production (GraphPad Software). A *P* value of <0.05 was considered significant.

## 3. Results

### 3.1 Histone H4 is released from the infarcted myocardium

**Figure 1:**
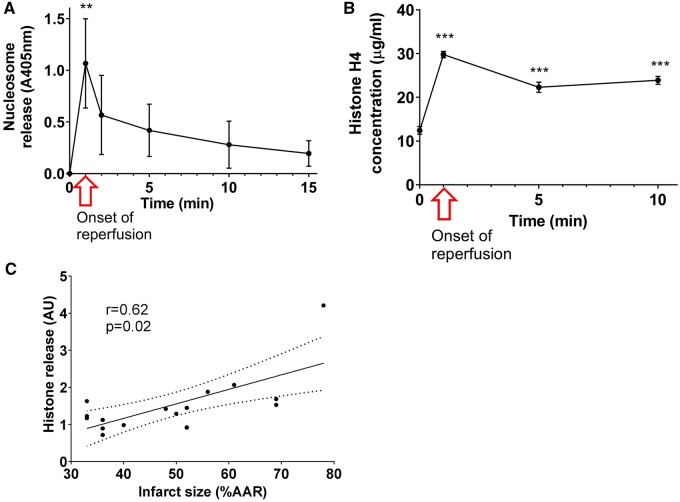
Nucleosomes and histone H4 are released from rat hearts subject to ischaemia and reperfusion. (*A*) Nucleosome concentration in the perfusate of perfused heart measured by ELISA (*N* = 5 hearts per group). (*B*) Histone H4 concentration in the perfusate of perfused heart measured by DELFIA. *N* = 8 hearts per group. (*C*) Total histone H4 release is correlated to final infarct size (*N* = 16). Statistical analyses by repeated measures ANOVA followed by Tukey test. **P < 0.01, ***P < 0.001 compared to the measurement taken before reperfusion.

Previous studies showing that the NLRP3 inflammasome and caspase 1 are upregulated during the process of MI,^12^^,^^24^ demonstrate that both cell necrosis and pyroptosis occur within ischaemic tissue. This type of cell death may release intracellular debris such as DNA and histones into the infarct.^4^ To test this hypothesis, Langendorff-perfused, isolated rat hearts were subjected to 45 min ischaemia followed by 2 h reperfusion. The concentration of nucleosomes in the perfusate, as measured by ELISA, was below detection levels prior to ischaemia, but rapidly increased to a maximum value immediately following the onset of reperfusion (*N* = 5, *P* < 0.01) (*Figure 1A*), then gradually decreased over 15 min. A specific assay for histone H4 also found a significant increase at reperfusion (*N* = 8, *P* < 0.01) (*Figure 1B*). The concentration of histone H4 in the perfusate of hearts following I/R injury reached a maximum of 29.8 ± 0.7 µg/mL. There was a positive correlation between the infarct size and the concentration of free unbound histone H4 in the perfusate released immediately at the onset of reperfusion (*Figure 1C*).

### 3.2 Histone toxicity to cardiomyocytes is prevented by mCBS or HIPe

**Figure 2:**
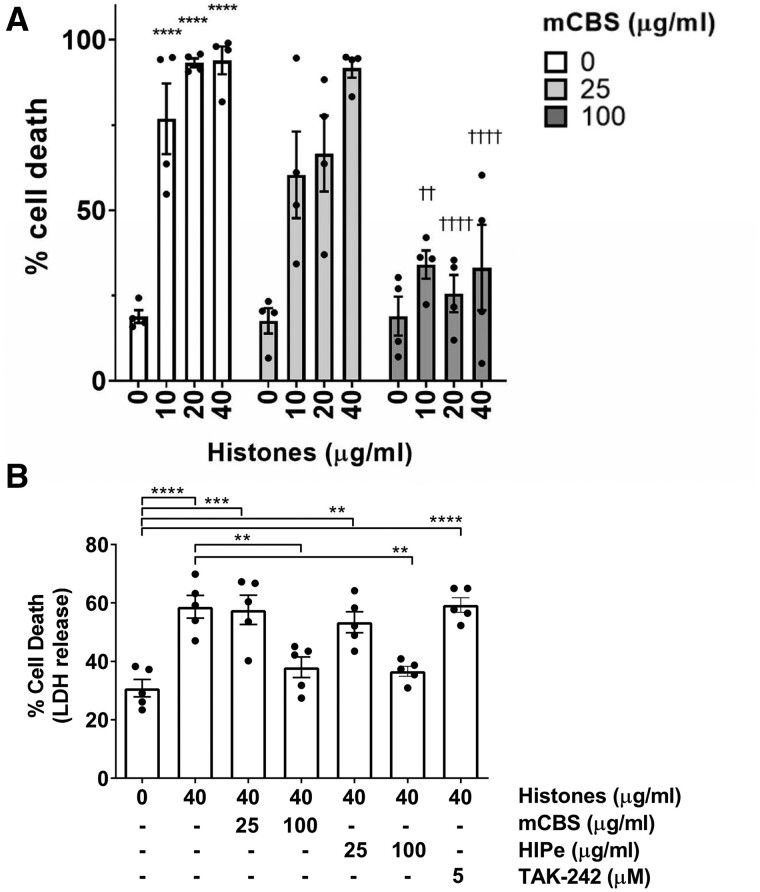
Histones exhibit a dose dependent cytotoxic effect on isolated rat cardiomyocytes *in vitro*, which can be reversed by the addition of mCBS. (*A*) Primary adult rat cardiomyocytes were incubated for 1 h with pure histones in the presence of 0, 25, or 100 μg/mL mCBS. The percentage of cell death was determined by PI staining. *N* = 4 independent biological experiments. Analysed by two-way ANOVA with Bonferroni correction for multiple comparison. ****P < 0.0001 vs. control group. ††*P* < 0.01 ††††*P* < 0.0001 vs. 40 μg/mL histones alone. (*B*) LDH release was used to assay cell death in primary cardiomyocytes treated with the drugs at concentrations indicated. *N* = 5 independent biological experiments. Analysis by one-way ANOVA and Tukey post-test. **P < 0.01, ***P < 0.001, ***P < 0.0001.

To establish whether histones are cytotoxic to cardiomyocytes, primary adult rat cardiomyocytes were incubated for 1 h with 0 to 40 µg/mL free histones derived from calf thymus, which contain a range of histones including histone H4. Cell survival was then assessed by staining with propidium iodide (PI). 10 µg/mL histones significantly increased cell death from 18.9 ± 1.9% to 76.8 ± 10.4% (*N* = 4, *P* < 0.001), while 20 and 40 µg/mL histones caused the death of almost all cells (*Figure 2A*). Similar results were seen when using an LDH assay to assess cell death although the overall level of death was lower (*Figure 2B*).

Treatment with 25 µg/mL mCBS did not offer any protection against histones as measured by PI staining (*Figure 2A*). However, 100 µg/mL mCBS significantly reduced cardiomyocyte death caused by 40 µg/mL of histone (33.7 ± 12.5% vs. 91.7 ± 2.8%, *P* < 0.0001, *N* = 4) (*Figure 2A*), and was also effective at reducing cell death caused by 10 μg/mL or 20 μg/mL histones (*Figure 2A*). Significant protection by 100 µg/mL of mCBS against 40 μg/mL histones was also seen when assayed by LDH release (*N* = 5, *P* < 0.01) (*Figure 2B*). 100 μg/mL but not 25 μg/mL HIPe was also able to prevent the cytotoxic effects of 40 μg/mL histones in the LDH assay (36.6% ± 3.8% vs. 58.7% ± 8.7%, *N* = 5, *P* < 0.01) (*Figure 2B*).

### 3.3 mCBS reduces infarct size in isolated rat hearts subject to I/R

**Figure 3:**
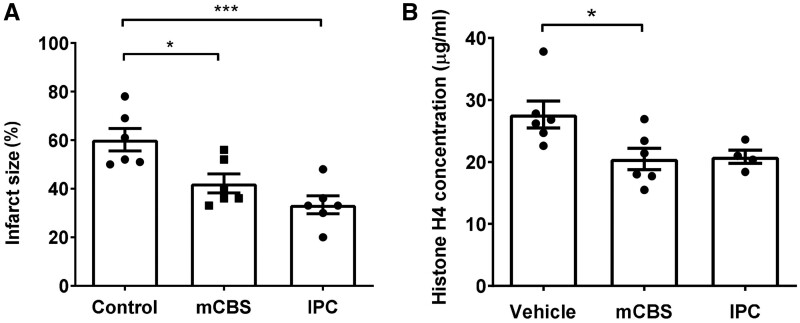
The histone neutralizing agent mCBS reduces infarct size in an *ex vivo* rat heart model of ischaemic and reperfusion injury (I/R). (*A*) Infarct size in hearts exposed to 45 min of global ischaemia followed by 2 h reperfusion, in the presence of vehicle or 100 μg/mL mCBS or pre-treated with ischaemic preconditioning (IPC). *N* = 6 hearts per treatment group. (*B*) The concentration of free histone H4 in the perfusate of rat hearts at the point of reperfusion after 45 min of ischaemia, measured by DELFIA. *N* = 6 (or 4 IPC) hearts per group. Statistical analyses by one-way ANOVA with Tukey post-test. *P < 0.05, ***P < 0.001.

Hearts isolated from anaesthetized rats were mounted on a Langendorff apparatus and subjected to 45 min of global ischaemia followed by 2 h reperfusion in the presence of mCBS or vehicle prior to I/R, then infarct size was measured. Perfusion of mCBS throughout I/R significantly reduced infarct size in comparison to vehicle (42.2 ± 9.5% vs. 60.2 ± 11.4%, *P* < 0.05, *N* = 6) (*Figure 3A*) The positive control of ischaemic preconditioning (IPC), consisting of 5 min ischaemia and 5 min reperfusion repeated 3 times, also reduced infarct size significantly (33.3 ± 9.0% vs. 60.2 ± 11.4%, *P* < 0.001, *N* = 6) (*Figure 3A*).

To confirm that mCBS was binding and sequestering histones, the perfusate was collected from the above experiments and analysed for free histone H4. Hearts that were treated with mCBS had significantly less histone H4 detectable in the perfusate (27.6 μg/mL ± 5.2 vs. 20.4 μg/mL ± 4.2, *n* = 6, *P* = 0.04) (*Figure 3B*). IPC also appeared to cause a reduction in histone H4 although this was not significant.

**Figure 4:**
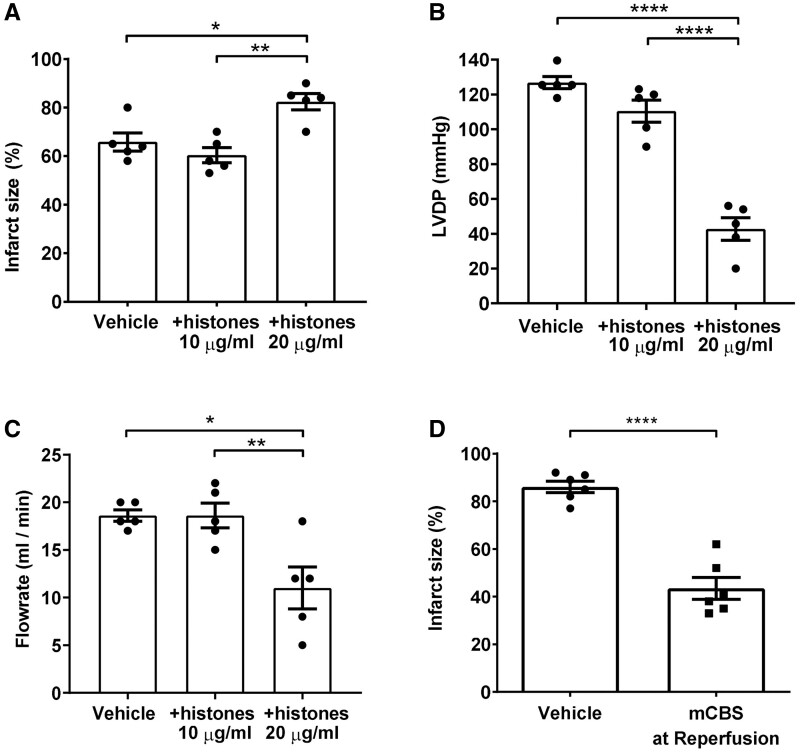
Extracellular histones contribute to myocardial infarction during reperfusion, and histone neutralization during reperfusion is cardioprotective. (*A–C*) Exogenous histones or vehicle were added to the perfusate of Langendorff-perfused rat hearts following 45 min ischaemia and during 2 h reperfusion. Infarct size (*B*) was measured at the end of the experiment. Left ventricular developed pressure (LVDP) (*B*) and flow rate of the perfusate (*C*) were measured 5 min into the reperfusion period. *N* = 5 hearts per group. (*D*) Infarct size in hearts subject to 45 min of global ischaemia followed by 2 h reperfusion, in the presence of vehicle or 100 μg/mL mCBS. *N* = 6 hearts per group. Statistical analyses by one-way ANOVA with Tukey post-test (*A–C*), or by unpaired *T*-test (*D*). *P < 0.05, **P < 0.01, ****P < 0.0001.

To ascertain whether excess histones exacerbate cardiac injury during reperfusion, free histones were added to the perfusate following 45 min ischaemia and during 2 h reperfusion. 10 μg/mL histones did not significantly affect infarct size at the end of the experiment (*Figure 4A*), or left ventricular end diastolic pressure (LVDP) or flow rate measured after 5 min reperfusion (*Figure 4B and C*, respectively). However, the addition of 20 μg/mL histones significantly increased infarct size from (65.8 ± 3.7% to 82 ± 3.3%, *n* = 5, *P* = 0.012) (*Figure 4A*). The increased damage to the myocardium was also reflected by a significant decrease in LVDP (127 ± 3.5 to 43 ± 6, *n* = 5, *P* < 0.0001) (*Figure 4B*) and flow rate (18.6 ± 0.6 to 11 ± 2.2, *n* = 5, *P* = 0.01) (*Figure 4C*).

To establish whether cardioprotection could be observed by inhibiting histones during reperfusion, isolated, perfused rat hearts were subject to 45 min of global ischaemia followed by 2 h reperfusion, as above, with the addition of mCBS during only the reperfusion period. This was also able to significantly reduce infarct size from 86.0 ± 2.4 to 43.5 ± 4.6, *n* = 6, *P* < 0.0001 (*Figure 4D*).

### 3.4 Infarct size in an in-vivo rat I/R model is reduced by HIPe but not mCBS

**Figure 5:**
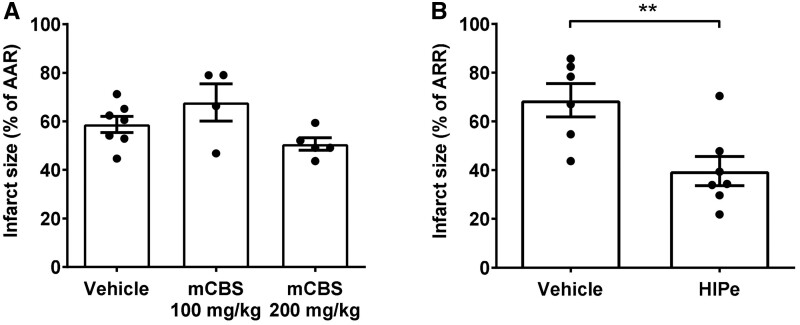
In an *in vivo* rat model of ischaemic and reperfusion injury, the histone-H4 specific neutralizing peptide HIPe, but not mCBS, reduced infarct size. (*A*) Infarct size as a percentage of area at risk (AAR) in an *in-vivo* rat coronary artery occlusion model of I/R, after administration of vehicle or mCBS. *N* = 4–7 hearts per group. (*B*) Infarct size as a percentage of AAR in an *in-vivo* rat coronary artery occlusion model of I/R, after administration of vehicle or HIPe. *N* = 6–7 hearts per group. Statistical analyses by one-way ANOVA with Tukey post-test (C), or unpaired *T*-test (D). **P < 0.01, ***P < 0.001.

To determine whether histone antagonists are effective *in vivo*, anaesthetised rats were subjected to 30 min coronary occlusion via suture ligation of the left anterior descending artery followed by 2 h reperfusion. 10 min prior to the onset of ischaemia the rats were administered vehicle (PBS) or mCBS (100 mg/kg) as a bolus via i.v. injection, or mCBS (200 mg/kg) divided equally as an initial i.v. bolus followed by infusion throughout I/R. The ischaemic area at risk (AAR) was similar in all groups (Supplementary material online, *Figure 1A and B*). mCBS did not significantly affect the infarct size *in vivo* in comparison to the vehicle (*Figure 5A*). In contrast to mCBS, HIPe (2 mg/kg), which is selective for histone H4, significantly reduced myocardial infarct size from 68.6 ± 6.8% to 39.6 ± 6.0% (*P* = 0.008, *Figure 5B*).

### 3.5 Histone-induced cell cytotoxicity occurs via a TLR-independent mechanism

Previously, histone H4 has been shown to stimulate TLR4.^25^ To investigate this, cardiomyocytes were incubated with 5 μM resatorvid (TAK-242), a selective TLR4 inhibitor that binds the intracellular domain of TLR4 and suppresses its signalling.^26^ However, this did not affect cell death cause by 40 μg/mL histones, as measured by LDH release (*Figure 2B*).

**Figure 6:**
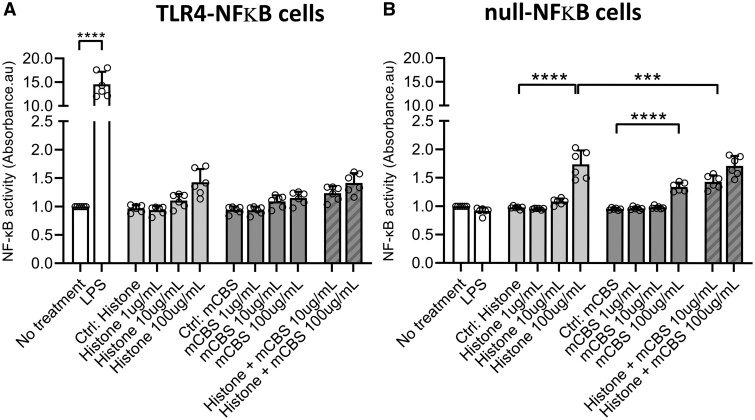
HEK293 reporter cells expressing a TLR4 reporter (*A*) or null control construct (*B*) were exposed to histones, mCBS or LPS overnight, then secreted embryonic alkaline phosphatase was measured as an index of reporter activity. The positive control of LPS caused a huge increase of NFκB activity as expected (*N* = 6 independent biological experiments) (*P* < 0.01) analysed by one-way ANOVA and Tukey post-test. A small increase in NFκB activity was seen at the highest concentration of 100 μg/mL histones, but as this occurred similarly in control cells, it was not related to TLR4 stimulation.

**Figure 7:**
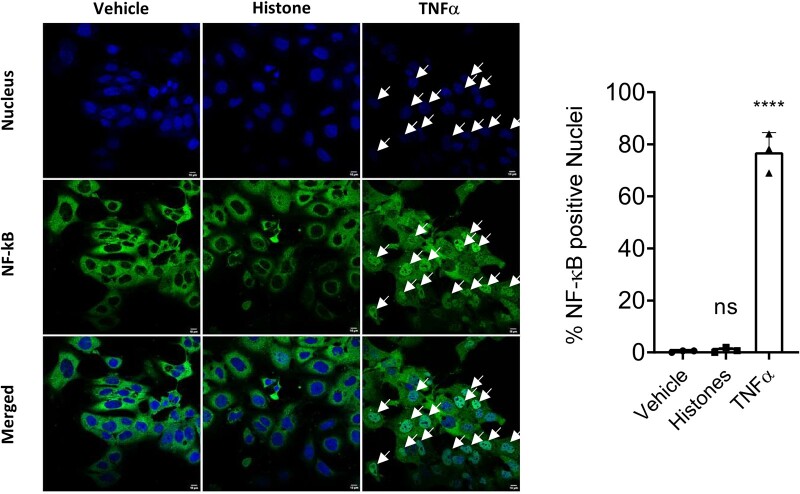
Histones do not cause activation and nuclear re-localization of NF-κB in HL-1 cardiomyocytes. HL-1 cells were treated with vehicle, 10 ng/mL TNFα or 10 μg/mL histones for 40 min, before fixation and staining of NF-κB. Only TNFα caused significant nuclear relocalization as shown in representative images and quantification (*N* = 3 experiments). Statistical analysis by one-way ANOVA with Tukey post-test. *=*P* < 0.0001; ns = non-significant, in comparison to vehicle.

To further investigate whether histones are capable of directly stimulating TLR4, a HEK293 reporter cell line expressing the TLR4, MD-2, and CD14 co-receptor genes was used. Stimulation with a TLR4 ligand induces the expression of the reporter gene as measured by the levels of secreted alkaline phosphatase. First, the toxicity of histones to these cells was determined using an MTT assay. Histone concentrations up to 10 µg/mL did not affect reporter cell viability but 100 μg/mL resulted in significantly increased cell death (Supplementary material online, *Figure S1C* and D). Co-incubation with mCBS prevented the cytotoxic effects of histones added to the HEK293 reporter cells (Supplementary material online, *Figure S1C and D*). Next, TLR4 activity was measured after treatment overnight. The positive control of LPS caused a large increase in TLR4-mediated NF-κB activation, as expected (*Figure 6A*). A small increase in NF-κB activity was seen at the highest concentration of 100 μg/mL of histones, but a similar effect was seen in control (null) cells lacking TLR4, indicating it was not related to TLR4 stimulation (*Figure 6A and B*). The inability of histones to stimulate TLR4-mediated NF-κB activation was confirmed in the HL-1 cardiomyocyte cell line. In contrast to TNFα, which caused clear NF-κB relocalization to the nucleus, histone treatment caused no NF-κB relocalization (*Figure 7*).

## 4. Discussion

In summary, we have shown that histone H4 and nucleosomes are released from rat hearts during global I/R. *In vitro* studies showed that histones are cytotoxic to primary adult rat cardiomyocytes, via a mechanism independent of TLR4 and NF-kB signalling. Importantly, neutralization of extracellular histones with the polyanion mCBS reduced infarct size in an isolated rat heart model of global I/R. mCBS also reduced infarct size when commenced at the onset of reperfusion. Furthermore, we have shown that selective inhibition of histone H4 with HIPe resulted in a significant reduction in myocardial infarct size *in vivo*. These data provide proof-of-principle that selective targeting of histone H4 may be a useful adjunct therapy to target I/R injury, either individually or in combination with other cardioprotective treatments.^27^

Histones have been shown to be toxic in a wide variety of pathologies. Extracellular histones have previously been shown to be cytotoxic to endothelial cells^10^ and kidney cells^8^ and have also been implicated in the pathogenesis of sepsis-induced cardiomyopathy.^28^ In humans, the maximum concentration of circulating histones clinically detected in an inflammatory disorder such as severe sepsis is between 200 and 400 μg/mL.^11^ Exposure to 50 µg/mL histones for 3 h was previously shown to be toxic to rat cardiomyocytes *in vitro.*^25^ Here, we show that exposure to just 10 µg/mL histones for only 1 h is sufficient to cause significant cardiomyocyte death *in vitro*. Addition of 20 µg/mL histones to the perfusate during reperfusion significantly increased infarct size in isolated hearts. In the perfusate from an isolated heart subject to I/R, we measured a maximum concentration of 29.8 ± 0.7 µg/mL histone H4. Since the releasate from damaged cardiomyocytes is diluted by the volume of the perfusate, the local concentration of histone H4 within the myocardium may potentially be much higher than this. These data indicate that the histone concentration achieved during I/R is sufficient to cause significant injury.

A positive correlation was observed between the infarct size and the concentration of free unbound histone H4 in the perfusate released immediately at the onset of reperfusion. This correlation was expected, given that, in the neutrophil-depleted Langendorff model, extracellular histone release only occurs upon cellular necrosis and rupture of the cell membrane.^29^

Both the compound mCBS and the peptide HIPe were able to protect against histone-induced cytotoxicity in the *in vitro* assay. Furthermore, mCBS was cardioprotective in an isolated, perfused heart model of IR, to a similar extent as IPC. mCBS was also cardioprotective when present only during reperfusion. There were lower levels of histone-H4 in the perfusate from hearts treated with mCBS, presumably because the infarcts were smaller and therefore less histone was released from dying cells. It was not feasible to perfuse hearts with HIPe due to the expense of the peptide that would be required.

Interestingly, heparin has previously been demonstrated to protect endothelial cells and the heart from the cytotoxic effects of histones, both *in vitro* and *in vivo.*^30^^,^^31^ The mechanism for histone’s cardioprotection has been suggested variously to be due to inhibition of complement; suppression of NF-κB signalling thereby inhibiting the release of TNF-α; or through a nitric oxide-cyclic guanosine monophosphate pathway.^30–32^ This leaves the role of histone binding by heparin uncertain. Heparin is certainly able to bind to histones,^33^ but its use for this purpose may be limited by its anti-coagulant effect at high doses. mCBS has the important advantage of lacking the pro-coagulant effect of heparin and can be given at higher doses which should make it more effective than heparin at neutralising histones.

In contrast to the *ex vivo* Langendorff experiments, mCBS did not significantly affect the infarct size following I/R *in vivo*. This indicates that mCBS may not be cardioprotective in the presence of blood at the doses used in the setting of I/R. We speculate that the reason for this is that the non-specific nature of the electrostatic binding means that mCBS may bind with greater affinity to other proteins in high abundance in blood, leaving insufficient amounts to sequester histones in an acute I/R model.^17^ However, in contrast, HIPe which is selective for histone H4, significantly reduced myocardial infarct size *in vivo*. It will be important in future experiments to address the question of whether HIPe is cardioprotective when administered at, or shortly after, reperfusion, which is the clinically relevant time-point in patients presenting with an acute MI.

The innate immune system appears to play a role in cardiac I/R injury,^34^ as TLR inhibitors reduce infarct size in animal models.^3^ TLR activation is also believed to play a crucial role in activating the intracellular inflammasome, a protein structure that causes cell death via pyroptosis, thereby allowing the release of IL-1β into the surrounding cellular matrix.^35^ Evidence for pyroptosis in I/R injury comes from studies with caspase-1 inhibitors, which reduce cardiac I/R injury ^36^ and the IL-1β inhibitor, Canakinumab, which in humans has shown to result in a 15% reduction in mortality associated with all cause cardiovascular disease.^37^

Previously, histone H4 has been shown to stimulate TLR4,^25^ however, increasing evidence suggests that it may act independently of TLRs.^10^ We found that resatorvid (TAK-242), a selective TLR4 inhibitor that binds the intracellular domain of TLR4 and suppresses its signalling,^26^ did not affect cell death caused by histones. Furthermore, histone H4 addition to a TLR4-reporter cell line did not cause any activation beyond a small amount of non-specific activation that was also seen in the control (null) cells lacking TLR4. These results were supported by experiments in HL-1 cardiomyocytes, in which histones failed to cause any nuclear translocation of NF-kB. Together, these results suggest that extracellular histones are not, in fact, DAMPs, as they do not cause TLR4-mediated activation of NF-kB, and histone-induced cardiomyocyte cell cytotoxicity occurs independently of TLR4 activation. In this regard, atomic force microscopy has been used to demonstrate that recombinant histone H4 is capable of causing cell membranes to bend, and directly causes pore formation.^10^ Small-angle X-ray scattering was used to show that the N-terminal domain of histone H4 causes a similar degree of membrane-remodelling of small unilamellar vesicles as other known membrane-remodelling proteins.^10^ Importantly, the HIPe peptide was shown to bind to the N-terminus of histone H4 and prevent histone H4 from interacting with and altering cell membranes.^10^ Taken together, the experiments shown here using TAK-242 and HIPe, and our previous experiments demonstrating that HIPe nullifies the pore-forming effect of histones, strongly indicate that the cytotoxic effect of histones is independent of TLR4.

It may be prohibitively expensive to synthesize HIPe in quantities sufficient to administer to humans. Nevertheless, our data encourage the development of small molecules to mimic its selective inhibition of histone H4 in the setting of I/R injury.

## Supplementary material

Supplementary material is available at *Cardiovascular Research* online.

## Authors’ contributions

S.M.D. and D.Y. designed research studies; M.S., A.R., S.B.K., S.A., Z.H., and C.M.H. conducted experiments; M.S., Z.H., A.R., S.B.K., S.A., C.M.H., K.O.S., S.A., S.M.D., and D.Y. analysed data; O.S. and C.R.P. provided reagents; M.S., S.M.D., and D.Y. wrote the manuscript; and all authors revised the manuscript.

## Supplementary Material

cvab139_Supplementary_Data
