# Supplementary material for: Extracellular histones are a target in myocardial ischaemia–reperfusion injury
Source: Cardiovasc Res. 2021 Apr 20;118(4):1115–25. doi: 10.1093/cvr/cvab139 (PMC8930072; doi:10.1093/cvr/cvab139)
Supplement: cvab139_Supplementary_Data [file cvab139_supplementary_data.docx]

**Extracellular histones are a target in myocardial ischaemia reperfusion injury**

Mohammed Shah, Zhenhe He, Ali Rauf, Siavash B Kalkhoran, Christina Mathisen Heiestad, Kåre-Olav Stensløkken, Christopher R Parish, Oliver Soehnlein, Sapna Arjun, Sean M Davidson, Derek Yellon

Supplementary information

# Supplementary Figure 1


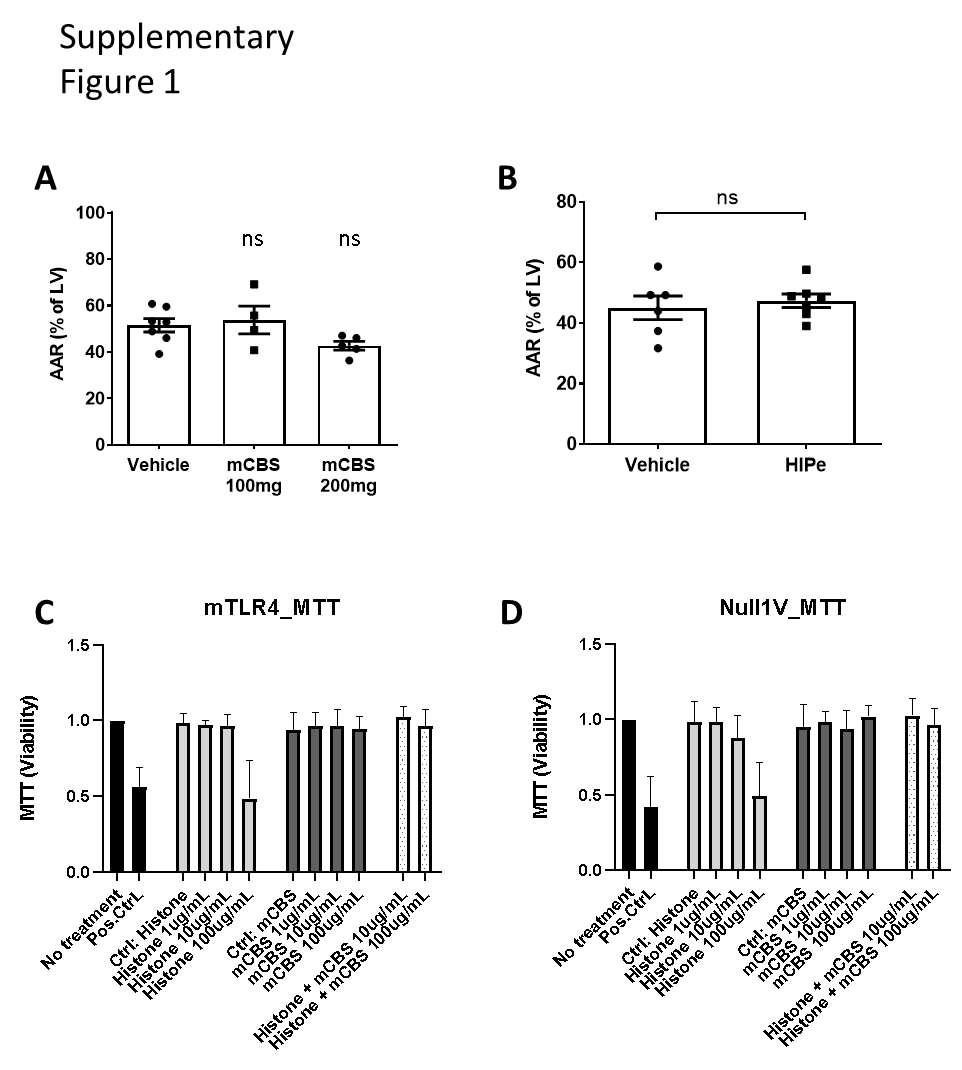


**A,B)** The area at risk (AAR) of the left ventricle (LV), in an *in-vivo* rat coronary artery occlusion model of I/R, after administration of vehicle or drugs as indicated. There was no significant difference (ns) between groups by 1 way ANOVA (N=7,4,5 in A; N=6,7 in B).

**C,D)** Survival of HEK293 cells treated as indicated, as measured by MTT assay. The positive control of LPS (Pos.Ctrl) and 100 μg/ml histones decreased cell viability, equally in cells expressing the TLR4 receptor (C) or null vector (D). There was no significant difference (ns) between groups by 1 way ANOVA (N=6 independent experiments).
